# Stress-Induced Cardiomyopathy: As a Diagnosis That Is Time Sensitive and Anticipative in Certain Individuals

**DOI:** 10.1155/2018/5243419

**Published:** 2018-12-05

**Authors:** Vishal Dhulipala, Prema Bezwada, Rashika Gottimukkula, Joseph Abboud

**Affiliations:** ^1^Department of Medicine, The Brooklyn Hospital Center, Academic Affiliate of the Icahn School of Medicine at Mount Sinai, Clinical Affiliate of the Mount Sinai Hospital, 121 DeKalb Avenue, Brooklyn, NY 11201, USA; ^2^Department of Cardiology, Mount Sinai Hospital, 1 Gustave Levy Pl, New York, NY 10029, USA; ^3^Department of Cardiology, Mount Sinai Beth Israel Hospital, 10 Nathan D Perlman Pl, New York, NY 10003, USA; ^4^Department of Cardiology, The Brooklyn Hospital Center, Academic Affiliate of the Icahn School of Medicine at Mount Sinai, Clinical Affiliate of the Mount Sinai Hospital, 121 DeKalb Avenue, Brooklyn, NY 11201, USA

## Abstract

Stress-induced cardiomyopathy, also known as Takotsubo cardiomyopathy, presents as an acute coronary syndrome. However, the physiology and pathogenesis are different. It is imperative to treat stress-induced cardiomyopathy as an acute coronary syndrome, and once diagnosed, it is imperative to assess and treat heart failure and wait for the impaired myocardial energetics to resolve. At times, the myocardial recuperation is quick that we miss the diagnosis of Takotsubo cardiomyopathy.

## 1. Introduction

Takotsubo is a rare stress-induced cardiomyopathy that mimics a myocardial infarction but is characterized by the absence of any coronary artery occlusion. Most patients present with the typical systolic apical ballooning, particularly of the left ventricle (LV). The very first case was described in the 1990s in Japan and today occurs in 1-2% of troponin-positive acute coronary syndromes or ST-segment elevation myocardial infarctions [1–3]. We describe a case of Takotsubo cardiomyopathy with an improvement in her ejection fraction in a few weeks.

## 2. Case Report

A 74- year -old female with a past medical history of asthma and hypertension presented to the Emergency Department with typical anginal chest pain, 10/10 in severity, midsternal, pressure like “someone sitting on my chest”, radiating to the left arm, and associated with an episode of vomiting five hours prior to arrival. She also reported several similar prior episodes with exertion that lasted for a few minutes, over the last four months but were less intense. She stated that she started to have the pain upon hearing about her granddaughter's diagnosis of cancer.

Her cardiac history was significant for an episode of similar chest pain approximately fifteen years ago. She had a coronary angiogram done which revealed normal coronaries. She also had episodes of chest pain five years ago and had an abnormal nuclear stress test followed by a coronary angiogram, which showed normal coronaries as well. On both these occasions, the patient was supposedly told she had a “temporary” disease.

**Figure 1:**
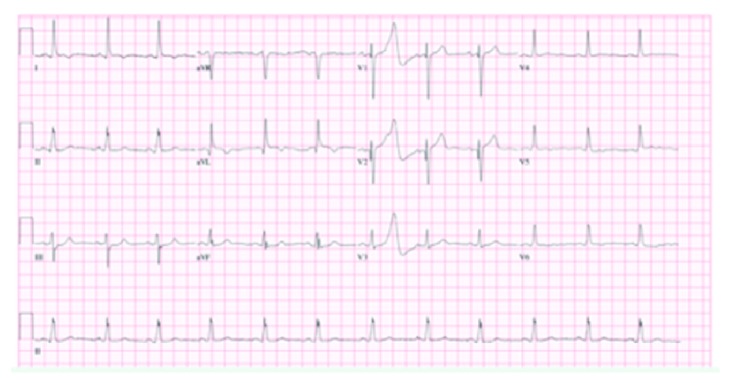
EKG representing T inversion in leads I and aVL.

**Figure 2:**
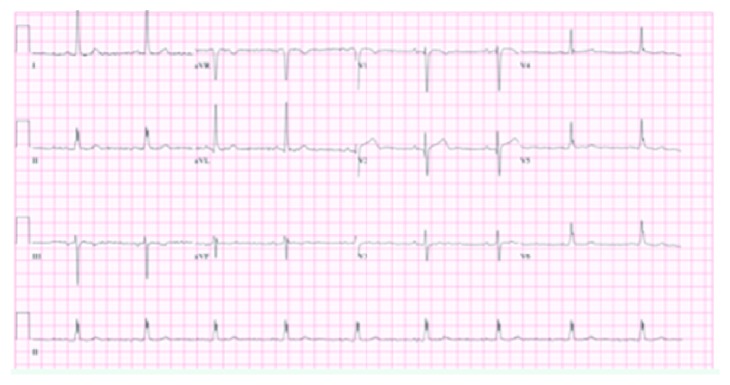
Old EKG without the T waves described in earlier EKG.

**Figure 3:**
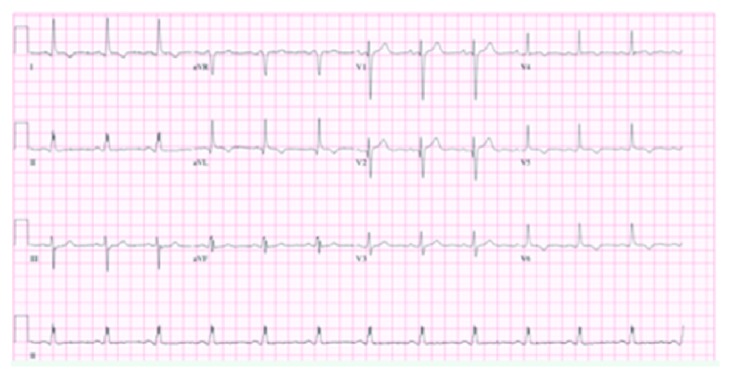
Repeat EKG showing dynamic T wave changes in leads I, aVL, V4, V5, and V6.

**Figure 4:**
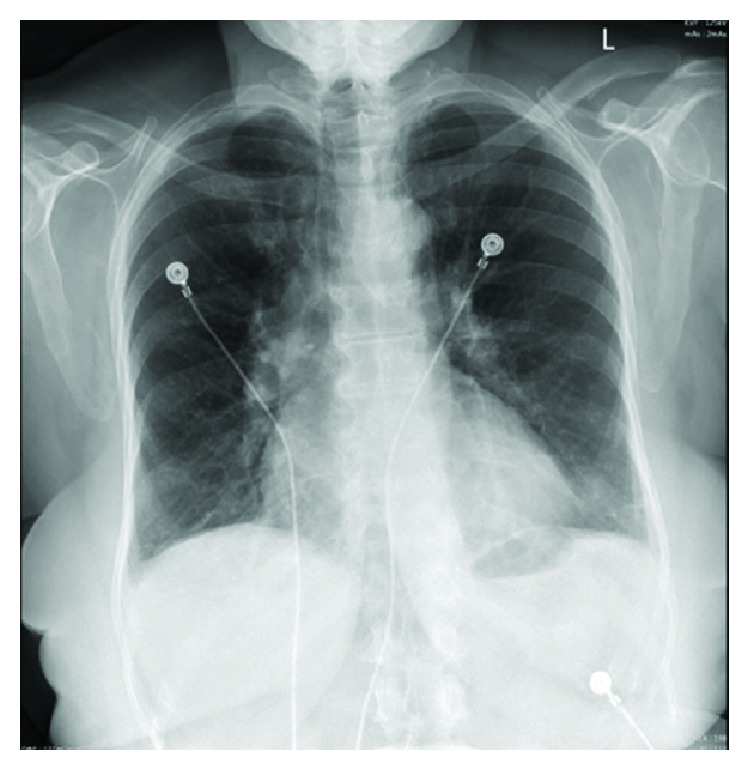
Chest X-ray showing cardiomegaly and pulmonary vascular prominence.

On admission, physical examination revealed an elderly woman in moderate distress due to pain with a blood pressure of 146/79 mmHg. Other vital signs were within the normal limit. Physical exam was also remarkable for bilateral basal crackles. Remainder of the physical exam was normal. Electrocardiogram (EKG) initially showed T wave inversions in leads I and aVL (Figure 1) when compared to her EKG a year ago (Figure 2). There were no ST elevations. A repeat EKG showed dynamic T wave changes with new T wave inversions in all the lateral leads (Figure 3). Chest X-ray (CXR) showed cardiomegaly, central pulmonary vascular prominence, and mild atelectasis over the lung bases (Figure 4). Labs including complete blood count and comprehensive metabolic panel were within normal limits. Troponin initially was 2.68 and peaked at 16.08. At admission, she received loading doses of dual antiplatelet therapy and was placed on a heparin drip for a non-ST elevation myocardial infarction (NSTEMI). On risk stratification, she had a total InterTAK diagnostic score of 61 points, which made her have a 58.6% chance of having Takotsubo cardiomyopathy.

A transthoracic echocardiogram revealed a left ventricle ejection fraction (LVEF) of 30-35%, moderate apical hypokinesis, and moderate mitral regurgitation with regional wall motion abnormalities. She was known to have a normal LVEF of 60-69% a year ago.

**Figure 5:**
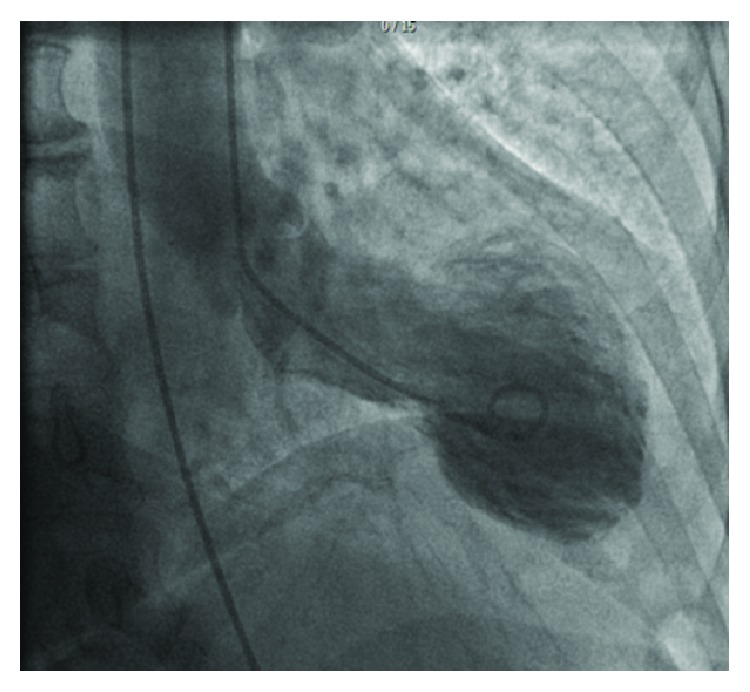
Cardiac catheterization with apical ballooning.

Cardiac catheterization showed normal coronaries with apical ballooning (Figure 5) and an elevated left ventricular end diastolic pressure (LVEDP). She was treated with aspirin 81 mg, warfarin, ACE inhibitor, beta blocker, and a moderate-intensity statin prior to discharge. A repeat transthoracic echocardiogram in 4 weeks revealed recovery of her left ventricular ejection fraction.

## 3. Discussion

A systematic review by Gianni et al. concluded that Takotsubo cardiomyopathy accounted for 1.7 to 2.2% of cases presenting as suspected acute coronary syndrome [2]. It occurs more commonly in postmenopausal women; as much as 9.9% were female with the mean age of 66.4 years, as reported in the International Takotsubo Registry [4]. The real pathogenesis has not been well understood. The most common explanations are catecholamine excess, coronary artery spasm, neurogenic stunning, and microvascular dysfunction, with catecholamine excess being the predominant theory [5, 6]. There is a catecholamine-induced microvascular spasm resulting in myocardial stunning, or it can cause direct catecholamine-associated myocardial toxicity [7]. It has also been reported that in some patients, the only visible stressor was exposure to catecholamines or beta-agonist drugs occurring in routine clinical doses [8]. Plasma catecholamines were measured at the onset of presentation [2, 9–11], and it was concluded that the plasma norepinephrine levels were elevated in 26 of 34 patients (74%) [2]. Rat models of immobilization-induced stress have also showed increased elevated catecholamines along with reversible LV ballooning [12].

Clinically, Takotsubo cardiomyopathy presents similar to acute coronary syndrome, along with elevated cardiac troponin levels in the majority of the cases. BNP or N-terminal pro-BNP has also been found to be elevated in most patients, up to as much as 82.9% as reported in the international Takotsubo Registry [4]. ST segment elevations in the EKG occur mainly in the anterior pericardial leads [13]. Initially, the diastolic function is similar to that of an acute myocardial infarction, but the systolic function can be similar or reduced [14, 15]. Echocardiographic features are predominantly left ventricular dysfunction due to akinesia of the midapical segments of the left ventricle (apical ballooning). Radionuclide myocardial perfusion imaging shows transient perfusion abnormalities, in the left ventricular apex. Other variants include mid and basal segment akinesia [11]. The in-hospital complication rates are very similar to those of acute coronary syndrome. Cardiogenic shock, invasive and noninvasive ventilation, cardiopulmonary resuscitation, and death occurred in 19.1% of patients as reported in the Takotsubo Registry versus 19.3% in a control group comprised of patients with acute coronary syndrome [4]. Patients tend to recover the ventricular systolic function within four weeks after the acute episode, with the mean LVEF which increased from 29% at the onset to 63% at an average of 6 days [16].

## 4. Conclusion

Our hypothesis here is that when our patient had chest pain in the past, she had minor catecholamine-induced myocardial stunning, which went unrecognized due to the early or quick recuperation of myocardial function ahead of the diagnostic imaging. Though a stress test was abnormal at one point, the cardiac catheterization did not show any coronary abnormalities. With that said, the real incidence of stress-induced cardiomyopathy may be higher than the one mentioned above. This will need higher anticipation by the physician and further study with a better use of the Takotsubo Registry.
